# Treatment of Obstructive Sleep Apnea May Slow Cognitive Decline in Older Adults

**DOI:** 10.1093/geroni/igaf122.4221

**Published:** 2025-12-31

**Authors:** Christopher Kaufmann, Jennifer Albrecht, Vishaldeep Sekhon, Halima Amjad, Emerson M Wickwire, Alden Gross, Atul Malhotra, Adam Spira

**Affiliations:** University of Florida, Gainesville, Florida, United States; University of Maryland, Baltimore, Maryland, United States; Johns Hopkins University School of Medicine, Baltimore, Maryland, United States; Johns Hopkins University School of Medicine, Baltimore, Maryland, United States; University of Maryland, Baltimore, Maryland, United States; Johns Hopkins Bloomberg School of Public Health, Baltimore, Maryland, United States; University of California, San Diego School of Medicine, La Jolla, Florida, United States; Johns Hopkins Bloomberg School of Public Health, Baltimore, Maryland, United States

## Abstract

Research links obstructive sleep apnea (OSA) to negative cognitive outcomes, but studies linking OSA treatment to cognitive decline have yielded inconsistent results. We investigated whether continuous positive airway pressure (CPAP) treatment was associated with cognitive performance over 10 years (2011-2021) among older adults with OSA. Data came from the National Health and Aging Trends Study (NHATS) linked with Medicare claims through the NIA LINKAGE Enclave. We identified N = 777 participants in the 2011 NHATS enrollment cohort who had >1 inpatient or outpatient claim with an OSA diagnosis (ICD-9=780.51, 327.23, 780.53, 327.23; ICD-10=G47.33, G47.30) and were free of cognitive impairment at baseline. CPAP treatment was defined as ≥ 1 claim for CPAP (HCPCS Code=E0601) at any time during study period. Cognitive performance was assessed using a factor-based total score derived from NHATS cognitive measures of executive function, orientation, and recall, modeled as a continuous outcome across study years. We fit generalized linear mixed models adjusting for age, BMI, education, and marital status, with interaction term for CPAP use by year. Cognitive scores declined over follow-up. In adjusted models, the annual decline among CPAP-treated participants was -0.03 factor score units per year (95% CI: -0.04, -0.02; p < 0.001). The CPAP*time interaction was -0.02 (95% CI: -0.04, -0.001; p = 0.040), indicating that participants without CPAP declined an additional 0.02 units per year, for an estimated decline of -0.05 units per year. CPAP treatment was associated with slower cognitive decline among older adults with OSA, and may thus represent a modifiable target to promote healthy brain aging.

